# Material and Administrative Components of Financial Toxicity and Survivorship Well‐Being: A Cross‐Sectional Analysis of HINTS (2012–2017)

**DOI:** 10.1002/cam4.71580

**Published:** 2026-01-30

**Authors:** Ivan H. C. Wu, Samuel R. Harris, Rachel Price, Shikha Bista

**Affiliations:** ^1^ Division of Epidemiology and Community Health, School of Public Health, University of Minnesota—Twin Cities Minneapolis Minnesota USA; ^2^ Masonic Cancer Center, University of Minnesota Minneapolis Minnesota USA; ^3^ College of Engineering and Applied Science, University of Colorado Boulder Boulder Colorado USA

**Keywords:** administrative burden, cancer survivorship, financial toxicity, Health Information National Trends Survey (HINTS), quality of life

## Abstract

**Background:**

Financial toxicity is a multidimensional construct shaped by material, psychological, and administrative pressures. This study examines how two components, namely, material burden and a narrow administrative indicator (insurance denial), are associated with cancer survivors' depression symptom severity and self‐perceived general health.

**Methods:**

We analyzed data from 959 cancer survivors in the Health Information National Trends Survey (HINTS; weighted *n* = 39,925,127). Material burden was measured by self‐reported financial harm due to cancer, and administrative burden by insurance denial. Depression symptom severity was assessed using the Patient Health Questionnaire‐4, and general health via self‐perceived health. Multivariate logistic and linear regression models evaluated associations between burdens and outcomes.

**Results:**

Nearly half (41.6%) of survivors reported financial harm from cancer, while 6.3% reported insurance denial. Material burden was significantly associated with increased depression symptom severity (odds ratio: 1.33; 95% CI: 1.01–1.70) and poorer self‐perceived health (*B*: −0.208; SE: 0.049; *p* < 0.001). Insurance denial showed no significant association with either outcome.

**Conclusion:**

Material burden was associated with survivors' mental and physical well‐being, underscoring the need for interventions that reduce financial strain. Insurance denial, while not directly associated with outcomes in this study, remains an important indicator of administrative burden. Future work should incorporate broader measures of administrative complexity to clarify its role in financial toxicity and survivorship outcomes.

## 1 Introduction

Financial toxicity refers to the negative financial impact that patients and their families experience because of cancer and the care it requires. Financial toxicity has gained increasing attention in recent years due to rising healthcare costs, the growing complexity of insurance systems, and the shift of financial responsibility to patients through higher copayments and deductibles. Direct out‐of‐pocket expenses (e.g., payments for visits, tests, and medications) and indirect costs (e.g., lost income, travel, and time required for care) undermine patients' financial stability and are widely recognized as contributors [1, 2]. The cumulative burden of medical and non‐medical costs can lead to long‐term economic hardship for individuals with cancer and their families, including an increased risk of bankruptcy [3, 4]. Older adults and individuals with limited financial resources are especially vulnerable because of reduced earning potential and reliance on fixed incomes [5]. Beyond material hardship, financial toxicity is also linked to psychological distress and worsened overall health [6].

Psychological and general physical health are robust indicators of survivorship and quality of life. Depression and anxiety are more common among cancer survivors than in the general population, with meta‐analyses estimating prevalence rates of 21%–37% and 18%–38%, respectively [7, 8, 9]. Rates of depression are highest among colorectal cancer patients (32%) [10] and are exacerbated by factors such as advanced disease stage, lower education levels, unemployment, and single status [8]. Mental health trajectories often worsen over time, highlighting the cumulative burden of cancer survivorship [11]. Furthermore, self‐perceived general health is linked to long‐term survival [12] and mortality risk [13]. Among breast cancer patients, for example, self‐rated health predicts both mortality and overall adjustment during survivorship [14, 15]. Survivors with higher comorbidity burdens and greater self‐management challenges report lower perceived general health, co‐occurring with poorer survival rates and quality of life [16], reflecting ongoing social inequities [12].

Although the terms “financial burden,” “financial hardship,” and “financial distress” are often used interchangeably, financial toxicity is a conceptually distinct framework that captures the material, psychological, and behavioral consequences of cancer‐related costs [17]. The material domain reflects the tangible strain caused by out‐of‐pocket expenses, medical debt, and lost income. The psychological domain involves distress, worry, and uncertainty associated with managing treatment costs. The behavioral domain includes changes in care‐seeking, such as delaying, modifying, or forgoing recommended care to cope with financial pressure. While these domains describe how financial toxicity is experienced and managed, cancer care also generates time and administrative demands (e.g., navigating insurance disputes or denials) that intersect with financial burden and compound the emotional strain of survivorship [18].

To address the multidimensional nature of financial toxicity and respond to calls for greater conceptual clarity [19], we draw on a framework that conceptualizes time, administrative, and financial burdens as interconnected facets of the survivorship experience [18]. This model, informed by the Burden of Treatment Theory [20] and the Cumulative Complexity Model [21], highlights how these burdens intersect to shape cancer survivorship. Time burdens reflect logistical demands of ongoing care such as appointment attendance and scheduling, and coordinating tasks that disrupt work and daily routines. Administrative burdens stem from navigating insurance systems and other bureaucratic processes, which can introduce delays, psychological stress, and barriers to treatment. Financial burdens include direct costs such as out‐of‐pocket spending and indirect costs such as medical debt and lost income. These domains interact; for example, insurance disputes can create delays that compound time losses and financial strain, intensifying distress and interrupting care [18, 19]. Burden of Treatment Theory further posits that when the workload of managing cancer care exceeds patients' capacity, whether due to administrative barriers, time demands, or financial pressure, it can erode emotional well‐being [20].

This study examines material and administrative burdens as distinct contributors to cancer survivorship outcomes, namely, depression risk and self‐perceived general health. While prior research documents the adverse impact of financial toxicity on health‐related quality of life [22], studies seldomly specify how different components of financial toxicity contribute to outcomes. By focusing on material burden and insurance denial, we hypothesize that higher material and administrative burdens will be associated with increased depression risk and lower self‐perceived general health.

## 2 Methods

### 2.1 Data

We used data from the Health Information National Trends Survey (HINTS) [23], a nationally representative, cross‐sectional survey of U.S. adults that collects self‐reported information on cancer‐related knowledge and behaviors. Sampling methods have been described elsewhere [23, 24]. Briefly, a stratified random sample of addresses was drawn, and one adult per household was selected. Data from three survey cycles were combined: HINTS 4 Cycle 2 (2012), HINTS 4 Cycle 4 (2014), and HINTS 5 Cycle 1 (2017). Analyses were restricted to respondents who reported a prior cancer diagnosis (*n* = 1510); cases with missing data on key variables were excluded (*n* = 551). This resulted in an unweighted sample of 959 cancer survivors (out of 10,592 total respondents). When weighted with replicate weights to account for the complex sampling design, the respondents represented an estimated population‐level count of 39,925,127 U.S. adults with a history of cancer, with each wave contributing approximately 20 million survivors to that total.

### 2.2 Measures

The primary outcomes were depression symptom severity and self‐perceived general health:

- *Depression symptom severity* was assessed using the Patient Health Questionnaire‐4 (PHQ‐4) [25], a validated tool for screening for anxiety and depressive disorders. PHQ‐4 scores range from 0 to 12. For analysis, scores were dichotomized into two categories: low depression severity (0–2) and moderate‐high depression severity (≥ 3).
- *General health* was measured with a single item: “In general, would you say your health is…,” scored on a 5‐point Likert scale. Scores were reverse‐coded such that higher scores indicated better health (1 = Poor health, 5 = Excellent health).

The primary exposure variables were derived from two items related to financial toxicity, conceptualized as material burden and administrative burden [18]:

- *Material burden* was assessed using a self‐reported measure of the negative financial effects of cancer and its treatment. Respondents rated their financial burden on a 4‐point scale (0 = Not at All, 1 = A Little, 2 = Some, 3 = A Lot), with the variable treated as continuous in the analysis.
- *Administrative burden* was based on a binary item indicating whether the respondent had ever been denied health insurance coverage due to their cancer (No/Yes). Administrative burden is operationalized using one HINTS item: insurance denial due to cancer. This indicator captures a concrete bureaucratic barrier within the healthcare system and represents the most feasible proxy for administrative burden available in the dataset. Although it reflects only one aspect of administrative work, prior studies show that insurance disputes can delay care, increase psychological stress, and intensify financial strain [18, 19].

In addition to controlling for survey year, covariates included the following demographic and clinical characteristics:

- *Sociodemographic characteristics* included Race/ethnicity (Hispanic, White, Black, Other), US region (Northeast, Midwest, South, West), education (Less Than High School, High School Diploma, Some College, College Graduate or Higher), household income (< $50,000, $50,000–$99,999, ≥ $100,000), marital status (Married vs. Not Married), urbanicity (Rural vs. Urban), insurance coverage (Yes/No), proportion of life spent in the United States (calculated as [Survey Year − Immigration Year]/Age, with US‐born coded as 1).
- *Cancer‐Related factors* included age at cancer diagnosis, number of treatment modalities (No Treatment, One Treatment, More than One Treatment).

### 2.3 Data Analysis

Analyses were conducted in R version 4.2.2 [26]. The ‘survey’ [27] and ‘srvyr’ [28] packages were employed to account for the complex sampling design and to produce population‐level estimates. The three HINTS waves (2012 HINTS 4 Cycle 2, 2014 HINTS 4 Cycle 4, and 2017 HINTS 5 Cycle 1)—each with 50 jackknife replicates—were merged into a single dataset following the recommended approach for combining independent samples [29]; this yielded 150 total replicate weights (50 per wave). The final weight from each respondent's respective survey was used, and each block of replicate weights activated variance contributions for only that wave. A jackknife replicate variance estimator with scale = 0.98 and rscales = 1 was applied to reflect the 50 replicates per wave and to adjust for stratification and clustering. Analyses were restricted to respondents who reported a prior cancer diagnosis, and individuals with missing data on key variables were excluded from the analytic models resulting in complete‐case analyses. Descriptive statistics (means, standard deviations, skewness, kurtosis) were calculated to assess data distribution. Two generalized linear models were then fitted: (1) a logistic model (quasibinomial) to examine depression symptom severity, and (2) a linear model (Gaussian) to examine general health. Two additional sensitive analyses were conducted recoding depression symptom severity as continuous (general linear model) and ordinal (ordinal regression) (see Tables S1 and S2). Variance inflation factors (VIFs) were computed to assess multicollinearity; cancer category was removed due to excessively high VIFs (58.17 for depression; 72.35 for general health), whereas the remaining covariates showed acceptable VIF levels (< 10 for depression; < 20 for general health). Residual analysis, including normal Q‐Q plots, supported the linear model assumptions for general health. Statistical significance was defined as *p* < 0.05.

## 3 Results

Table 1 presents the demographic characteristics of the sample. A majority of respondents (82%) self‐identified as white, followed by Hispanic (9%) and Black (6.1%) participants. Most respondents were female (58.4%), from the South (37.1%) and urban areas (78.3%). Socioeconomic indicators showed that most survivors reported household incomes below $50,000 (41.4%), most attended at least some college (72.9%), and a majority had health insurance coverage (95.4%). Most survivors reported receiving at least one cancer treatment (87.7%). In our sample, 13% of respondents reported no cancer treatment, likely due to early‐stage disease, watchful waiting, treatment refusal, or access barriers. Regarding health outcomes, almost a third of survivors (32%) were at mild, moderate, or severe symptoms of depression, and 22.6% reported fair or poor general health. Table 2 displays the distribution of the financial and administrative burdens variables. Most survivors reported no financial burden (58.3%) or being denied insurance coverage (93.7%) due to a cancer diagnosis.

**TABLE 1:** Sample demographic characteristics.

	Unweighted *n* (weighted %)
Race	
White	756 (82.0%)
Hispanic	82 (9.0%)
Black	84 (6.1%)
Other	37 (2.9%)
Sex	
Male	393 (41.6%)
Female	566 (58.4%)
Region	
Northeast	152 (16.9%)
Midwest	181 (22.0%)
South	393 (37.1%)
West	233 (24.0%)
Education	
< High school	64 (7.5%)
High school	182 (19.6%)
Some college	312 (40.9%)
College graduate	401 (32.0%)
Income	
< $50,000	472 (41.8%)
$50,000–$99,999	286 (34.1%)
> $99,999	201 (24.1%)
Health insurance	
Yes	910 (95.5%)
No	49 (4.5%)
Cancer treatments	
None	134 (13.6%)
One	555 (58.0%)
Multiple	270 (28.4%)
Urbanicity	
Urban	803 (78.4%)
Rural	156 (21.6%)
Marital status	
With spouse	516 (66.9%)
Without spouse	443 (33.1%)
Age (mean years)	64.67
Diagnosis age (mean years)	51.98
Time since diagnosis (mean years)	12.69
% time spent in the US	97%
Depression symptom	
Low	656 (68.0%)
Mild, moderate, severe	303 (32.0%)
General health	
Poor	47 (4.3%)
Fair	179 (18.3%)
Good	334 (35.4%)
Very good	314 (34.1%)
Excellent	85 (8.0%)

*Note:* Unweighted sample size was 959 cancer survivors. Weighted estimates accounting for complex sampling design represent an estimated 39,925,127 U.S. adults with a history of cancer.

**TABLE 2:** Financial and administrative burdens due to cancer diagnosis unweighted and weighted frequencies.

	Unweighted *n* (weighted %)
Denied coverage cancer	
Yes	54 (6.3%)
No	905 (93.7%)
Hurt finances cancer	
Not at all	564 (58.4%)
A little	170 (18.4%)
Some	123 (12.6%)
A lot	102 (10.5%)

A multivariate logistic regression analysis (Table 3) tested the hypothesis that greater financial toxicity is associated with increased depression symptom severity. Results supported our hypothesis. A one‐unit increase in self‐reported financial harm due to cancer was associated with a 32% higher likelihood of experiencing mild to severe depression (95% CI: 1.02, 1.69). Additionally, women were 73% more likely to report a higher symptom severity of depression compared to men (95% CI: 1.07, 2.79). No other covariates were significantly associated with depression symptom severity.

**TABLE 3:** Logistic regression for financial and administrative burden predicting depression symptom severity.

	Est.	SE	*t*‐value	*p*	OR	95% CI
Race (white reference)						
Hispanic	0.991	0.537	1.847	0.076	2.694	(0.894, 8.117)
Black	0.511	0.434	1.175	0.251	1.666	(0.682, 4.069)
Other	0.098	0.585	0.168	0.868	1.103	(0.332, 3.671)
Sex (male v. female)	0.546	0.233	2.344	0.027	1.726	(1.070, 2.785)
Region (northeast reference)						
Midwest	0.294	0.345	0.852	0.402	1.342	(0.660, 2.728)
South	0.278	0.308	0.903	0.375	1.321	(0.701, 2.489)
West	0.143	0.363	0.395	0.696	1.154	(0.548, 2.433)
Education (< HS reference)						
High school diploma	−0.251	0.545	−0.461	0.649	0.778	(0.254, 2.386)
Some college	−0.434	0.474	−0.915	0.369	0.648	(0.244, 1.718)
College graduate	−0.590	0.539	−1.095	0.283	0.554	(0.183, 1.678)
Income (< $50,000 reference)						
$50,000–$99,999	−0.621	0.316	−1.964	0.060	0.537	(0.280, 1.029)
> $99,999	−0.436	0.406	−1.074	0.293	0.647	(0.281, 1.489)
Health insurance (yes v. no)	1.266	0.770	1.644	0.112	3.548	(0.728, 17.278)
Cancer treatments (none reference)						
One	−0.236	0.414	−0.571	0.573	0.790	(0.338, 1.848)
Multiple	−0.344	0.439	−0.785	0.440	0.709	(0.288, 1.746)
Urbanicity (urban v. rural)	0.137	0.285	0.481	0.635	1.147	(0.639, 2.059)
Marital status (married v. unmarried)	0.101	0.268	0.377	0.709	1.106	(0.638, 1.918)
Age at diagnosis (years)	0.009	0.009	0.919	0.367	1.009	(0.989, 1.028)
Survey year (2012 reference)						
2014	−0.421	0.245	−1.714	0.099	0.657	(0.396, 1.088)
2017	0.026	0.259	0.102	0.920	1.027	(0.603, 1.750)
Proportion of life in US (%)	−0.559	1.257	−0.444	0.660	0.572	(0.043, 7.579)
Denied insurance coverage (yes v. no)	0.267	0.349	0.763	0.452	1.305	(0.637, 2.677)
Cancer Hurt finances (yes v. no)	0.274	0.123	2.235	0.034	1.316	(1.022, 1.693)

A multivariate linear regression model (Table 4) tested the hypothesis that greater financial and administrative burden is associated with worse self‐perceived general health. Results supported our hypothesis. Cancer survivors who reported financial harm due to cancer treatment reported significantly worse general health perceptions (*b* = −0.212, SE = 0.050, *p* < 0.001). Higher income levels were associated with better general health perceptions, with respondents earning $50,000–$99,999 (*b* = 0.435, SE = 0.100, *p* < 0.001) and those earning over $99,999 (*b* = 0.386, SE = 0.127, *p* = 0.005) reporting significantly better general health compared to those earning less than $50,000. Additionally, college graduates reported better general health than individuals with less than a high school education (*b* = 0.391, SE = 0.186, *p* = 0.046).

**TABLE 4:** Linear regression for financial and administrative burden predicting general health.

	*b*	SE	*t* value	*p*
Race (white reference)				
Hispanic	−0.181	0.180	−1.005	0.324
Black	−0.156	0.155	−1.003	0.325
Other	−0.323	0.185	−1.751	0.092
Sex (male v. female)	−0.077	0.086	−0.903	0.375
Region (northeast reference)				
Midwest	−0.121	0.116	−1.039	0.309
South	−0.071	0.122	−0.581	0.567
West	−0.116	0.142	−0.817	0.421
Education (< HS reference)				
High school diploma	0.050	0.196	0.255	0.801
Some college	0.179	0.174	1.032	0.312
College graduate	0.391	0.186	2.100	0.046
Income (< $50,000 reference)				
$50,000–$99,999	0.435	0.100	4.371	< 0.001
> $99,999	0.386	0.127	3.028	0.005
Health insurance (yes v. no)	0.141	0.288	0.488	0.629
Cancer treatments (none reference)				
One	0.114	0.156	0.736	0.468
Multiple	0.107	0.186	0.574	0.571
Urbanicity (urban v. rural)	−0.118	0.095	−1.253	0.221
Marital status (married v. unmarried)	0.047	0.093	0.501	0.620
Age at diagnosis (years)	−0.005	0.003	−1.746	0.093
Survey year (2012 reference)				
2014	−0.108	0.103	−1.05	0.303
2017	−0.077	0.118	−0.657	0.517
Proportion of life in US (%)	−0.013	0.287	−0.046	0.964
Denied insurance coverage (yes v. no)	−0.088	0.142	−0.621	0.540
Cancer hurt finances (yes v. no)	−0.212	0.050	−4.279	< 0.001

## 4 Discussion

The purpose of this study was to examine the association between material and administrative components of financial toxicity and well‐being among cancer survivors. Using a nationally representative sample of U.S. cancer survivors (weighted *n* ≈ 40 million), we found that material burden (i.e., negative financial effects of cancer and its treatment) was significantly associated with depression risk and lower self‐perceived general health, whereas administrative burden (narrowly measured as insurance denial due to cancer) was not associated with these outcomes. Specifically, material burden was linked to a 1.33‐fold increase in depression risk and poorer general health, even after accounting for administrative burden. This pattern aligns with Parsons et al. [18], who emphasize that different forms of burden (time, financial, administrative) can independently and jointly affect survivor well‐being. Consistent with the Burden of Treatment Theory and the Cumulative Complexity Model, persistent and cumulative stressors (e.g., ongoing financial strain) are more likely to exceed patient capacity and impair mental and physical health than a single administrative disruption such as an insurance denial.

These findings are consistent with prior research documenting the association between material components of financial toxicity and adverse mental and physical health outcomes [30, 31, 32]. Financial strain, including out‐of‐pocket costs and income loss, has been shown to negatively impact the quality of life for cancer survivors [33, 34, 35, 36]. However, our study did not find an association between administrative burden, as measured by insurance denial, and well‐being. In this sample, 4% of survivors reported an insurance denial, consistent with national BRFSS estimates [37]. While insurance denial might intuitively affect well‐being, its narrow and distal nature as a single administrative event may limit its direct observable impact, especially compared with the continuous pressures of material burden. Survivors may also use compensatory strategies, such as appealing denials, switching plans, seeking charity care, or relying on social support, to buffer the immediate effects of insurance denial, reducing its measurable association with depression or general health.

It is also possible that the type and adequacy of a survivor's insurance coverage shape well‐being more profoundly than a single denial event [38]. Broader forms of administrative complexity such as high‐deductible plans, limited‐network policies, or plans with poor coverage for supportive services may generate sustained strain that exceeds the impact of any isolated denial. Subjective financial distress often arises from navigating complex reimbursement procedures, delayed approvals, prior authorizations, high upfront costs, or inadequate coverage [39, 40]. These administrative tasks reflect the “work” of managing cancer, which the Burden of Treatment Theory suggests can accumulate and overwhelm patient capacity even without discrete disruptive events. Emotional strain may also depend on survivors' access to adaptive resources such as financial reserves, social support, or insurance literacy. Reliance on family or social networks for assistance can ease or worsen distress, depending on whether the support feels burdensome [40]. This broader context aligns with international evidence showing that financial toxicity persists across diverse health systems including Canada, Australia, the UK, and China whenever administrative complexity or inadequate coverage delays care or shifts costs onto patients [33].

Conceptually, our results align with prior models outlining the consequences of financial and administrative burdens due to cancer [19]. While our study was cross‐sectional, financial burdens have the potential to trigger feedback loops where increased symptom burden reduces earning capacity, employment, and economic resources, thereby exacerbating financial strain and worsening physical and psychological symptoms [41]. Survivors employ strategies to stabilize their economic resources, but the effectiveness of these strategies depends on preexisting economic reserves, social insurance entitlements, and support networks. Resilient care resources play a crucial role in counteracting these pressures, particularly for survivors with limited financial resources or lower education levels, who are disproportionately affected by cancer‐related financial burdens [42]. These burdens not only impact patients but also have spillover effects on caregivers, further diminishing quality of life [43, 44]. Thus, financial toxicity functions as a structural mechanism that reinforces systemic inequities in healthcare, a perspective articulated explicitly in more recent models [17] [18, 19]. Rather than being passive consequences of resource scarcity, these burdens erode survivors' economic reserves, diminish their ability to navigate healthcare systems, and amplify the emotional and physical toll of cancer survivorship. While administrative burdens, such as insurance claim denials, may be buffered by compensatory behaviors or social support, financial burdens impose direct and immediate pressures that can perpetuate disparities in mental and physical health that actively generate cycles of vulnerability. In this light, while financial toxicity represents economic strain, it more aptly reflects structural forces that reorganize survivors' lives, systematically reinforcing disparities and embedding health inequities within healthcare systems.

### 4.1 Limitations

This study has important limitations. First, the cross‐sectional design prevents causal inference, and associations may reflect bidirectional relationships between burden and health. Second, because the HINTS dataset captures administrative burden through a single item (insurance denial due to cancer), our measure reflects a narrow facet of administrative burden and may underestimate its broader impact. Third, because the sample includes only individuals who survived long enough to participate, survivor bias may underestimate the magnitude of financial toxicity, particularly among those with advanced disease, severe toxicity, or limited resources. Fourth, all measures are self‐reported and subject to recall or social desirability bias. Finally, unmeasured factors such as caregiving demands, treatment intensity, or local resource availability may also influence burden and well‐being.

### 4.2 Implications

The economic burden experienced by cancer survivors and their families and its association with mental health and self‐perceived general health outcomes is well‐documented. However, there is a need to examine and implement interventions to address financial burdens at institutional and policy levels. At the policy level, China's government price negotiation and reimbursement policy has improved patient access to treatment and reduced disparities in insurance coverage [45]. Countries with stronger financial protection policies such as limits on out‐of‐pocket costs, targeted cancer‐specific assistance programs, and publicly funded supportive services show lower levels of financial hardship among patients, reflecting the role of structural design in shaping equity [46]. In the United States, state‐level Medicaid expansions under the Affordable Care Act have increased health insurance coverage, improved access to care, and reduced financial strain for low‐income adults [47]. Additional policies, such as those focused on price transparency, protections against surprise billing, and paid sick leave, have potential to reduce medical financial hardship [48]. Despite these advancements, Medicaid expansion remains limited to select states, costs remain high even for insured individuals, and the broader impacts of institutional policies require further investigation. These gaps highlight the need for coordinated structural reforms that directly reduce the material components of financial toxicity.

At the organizational level, emerging programs demonstrate potential but also highlight significant gaps. A pilot program at an academic medical clinic employing lay health workers and social workers to assess financial toxicity showed reduced financial burden and high feasibility and acceptability [49]. However, the sample receiving the program includes 65% of participants identifying as white and 75% reporting annual household incomes exceeding $100,000. These findings underscore the need to expand such programs to underrepresented communities, including lower‐income and minority populations, who are particularly vulnerable to financial toxicity. Health systems could operationalize these efforts by embedding financial navigation into routine oncology care, integrating screening tools for material and administrative burden, and linking survivors with social workers, legal support, or insurance specialists earlier in the care trajectory [50].

Community‐based approaches, such as clinician referrals to resources addressing the direct and indirect costs of cancer care [51] and legal and financial education programs [52], offer additional avenues for alleviating financial burdens among cancer survivors. However, these interventions often face limitations in reducing overall financial burden. For example, a pilot financial navigation program leveraging local community patient advocates and financial education partners reported decreased patient anxiety but failed to significantly reduce financial strain [53]. These findings suggest that while patient‐level programs provide incremental relief, they are insufficient without broader system‐level reforms. Effective and sustainable reductions in financial toxicity will require multi‐level strategies: federal policies to stabilize insurance coverage and reduce cost‐sharing, organizational changes that integrate financial navigation into standard care, and community partnerships that address the social and administrative barriers patients face.

## 5 Conclusion

This study shows that material components of financial toxicity are strongly associated with depression risk and poorer self‐perceived health among cancer survivors, while a single administrative barrier such as insurance denial was not. These findings indicate that persistent financial strain exerts more immediate effects on well‐being than isolated administrative events, though broader administrative complexities still merit investigation. The study contributes to ongoing calls [54] to clarify the conceptual structure of financial toxicity by empirically distinguishing material and administrative burdens and by demonstrating their differential associations with key survivorship outcomes. The results support policy and institutional strategies to reduce financial strain and improve equitable access to care and align with financial toxicity, [55] and support treatment burden frameworks [18] that highlight the imbalance between patient workload and capacity. Future work should use longitudinal designs to capture how financial toxicity evolves over time and refine measurement approaches that more fully characterize both material and administrative components of this burden.

## Author Contributions

**Ivan H. C. Wu:** conceptualization (lead), formal analysis (supporting), methodology (lead), supervision (lead), writing – original draft (lead), writing – review and editing (equal). **Samuel R. Harris:** data curation (lead), formal analysis (lead), methodology (supporting), writing – original draft (equal), writing – review and editing (equal). **Rachel Price:** writing – original draft (equal), writing – review and editing (supporting). **Shikha Bista:** writing – original draft (equal), writing – review and editing (equal).

## Funding

Ivan HC Wu is supported by the National Institutes of Health (R00MD015296 and P30CA077598). These funding sources had no role in the study.

## Conflicts of Interest

The authors declare no conflicts of interest.

## Supporting information


**Data S1:** cam471580‐sup‐0001‐Supinfo.docx.

## Data Availability

The data underlying this article are derived from publicly available datasets. Specifically, the data were obtained from the Health Information National Trends Survey (HINTS), accessible at https://hints.cancer.gov/. All relevant data and details about survey design, sampling, and methodology can be found on the HINTS website. Any additional data analysis scripts used in this study are available upon request from the corresponding author.
