# Supplementary material for: Material and Administrative Components of Financial Toxicity and Survivorship Well‐Being: A Cross‐Sectional Analysis of HINTS (2012–2017)
Source: Cancer Med. 2026 Jan 30;15(2):e71580. doi: 10.1002/cam4.71580 (PMC12856721; doi:10.1002/cam4.71580)
Supplement: Supplementary file 1 — Data S1: cam471580‐sup‐0001‐Supinfo.docx. [file CAM4-15-e71580-s001.docx]

Sensitivity analyses were performed for depression risk, treating the outcome, PHQ-4, as both continuous and categorical to ensure the results from logistic regression are robust. A linear regression model (Supplemental Table 1) was fitted with continuous PHQ-4 scores ranging from 0 to 12. The significance of the primary analysis did not change.

An ordinal regression model (Supplemental Table 2) for depression risk was also fitted, treating PHQ-4 score as a three-level categorical variable (no depression: 0-2, mild depression: 3-5, moderate/severe depression: 6-12). Estimates from the logistic regression model were used as starting value coefficients and -1 and 1 were chosen as intercepts. The significance of the primary analysis did not change.

| **Supplemental Table 1.** Linear regression for administrative and financial burden predicting depression risk. | | | | |
| --- | --- | --- | --- | --- |
|  | ***b*** | ***Std. Error*** | ***t value*** | ***p*** |
| **Race (White Reference)** |  |  |  |  |
| Hispanic | 1.225 | 0.771 | 1.589 | 0.124 |
| Black | 0.412 | 0.605 | 0.682 | 0.501 |
| Other | -0.222 | 0.585 | -0.380 | 0.707 |
| **Sex (Male v. Female)** | 0.595 | 0.268 | 2.221 | 0.035 |
| **Region (Northeast Reference)** |  |  |  |  |
| Midwest | 0.069 | 0.404 | 0.171 | 0.866 |
| South | 0.377 | 0.372 | 1.011 | 0.321 |
| West | 0.059 | 0.381 | 0.156 | 0.877 |
| **Education (< HS Reference)** |  |  |  |  |
| High School Diploma | 0.298 | 0.802 | 0.372 | 0.713 |
| Some College | -0.176 | 0.720 | -0.244 | 0.809 |
| College Graduate | -0.430 | 0.730 | -0.588 | 0.561 |
| **Income (< $50,000 Reference)** |  |  |  |  |
| $50,000 - $99,999 | -0.680 | 0.431 | -1.577 | 0.127 |
| >$99,999 | -0.615 | 0.411 | -1.493 | 0.148 |
| **Health Insurance (Yes v. No)** | 1.540 | 1.425 | 1.081 | 0.290 |
| **Cancer Treatments (None Reference)** |  |  |  |  |
| One | -0.615 | 0.519 | -1.185 | 0.247 |
| Multiple | -0.630 | 0.578 | -1.091 | 0.285 |
| **Urbanicity (Urban v. Rural)** | 0.281 | 0.445 | 0.632 | 0.533 |
| **Marital Status (Married v. Unmarried)** | 0.236 | 0.291 | 0.813 | 0.424 |
| **Age at Diagnosis (Years)** | 0.002 | 0.012 | 0.132 | 0.896 |
| **Survey Year (2012 Reference)** |  |  |  |  |
| 2014 | -0.391 | 0.282 | -1.386 | 0.178 |
| 2017 | -0.004 | 0.352 | -0.011 | 0.991 |
| **Proportion of Life in US (%)** | 0.708 | 1.106 | 0.641 | 0.527 |
| **Denied Insurance Coverage (Yes v. No)** | 0.612 | 0.542 | 1.129 | 0.269 |
| **Cancer Hurt Finances (Yes v. No)** | 0.429 | 0.169 | 2.540 | 0.017 |

| **Supplemental Table 2.** Ordinal regression for administrative and financial burden predicting depression risk. | | | | | |
| --- | --- | --- | --- | --- | --- |
|  | ***Estimate*** | ***Std. Error*** | ***t value*** | ***OR*** | ***95% CI*** |
| **Race (White Reference)** |  |  |  |  |  |
| Hispanic | 1.149 | 0.596 | 1.928 | 3.154 | (0.981, 10.138) |
| Black | 0.378 | 0.397 | 0.952 | 1.459 | (0.670, 3.173) |
| Other | 0.162 | 0.541 | 0.299 | 1.176 | (0.407, 3.396) |
| **Sex (Male v. Female)** | 0.532 | 0.237 | 2.240 | 1.702 | (1.069, 2.710) |
| **Region (Northeast Reference)** |  |  |  |  |  |
| Midwest | 0.154 | 0.347 | 0.445 | 1.167 | (0.591, 2.303) |
| South | 0.213 | 0.333 | 0.640 | 1.238 | (0.644, 2.378) |
| West | 0.077 | 0.362 | 0.212 | 1.080 | (0.532, 2.194) |
| **Education (< HS Reference)** |  |  |  |  |  |
| High School Diploma | 0.011 | 0.524 | 0.021 | 1.011 | (0.362, 2.826) |
| Some College | -0.251 | 0.442 | -0.569 | 0.778 | (0.327, 1.850) |
| College Graduate | -0.452 | 0.464 | -0.974 | 0.636 | (0.256, 1.580) |
| **Income (< $50,000 Reference)** |  |  |  |  |  |
| $50,000 - $99,999 | -0.512 | 0.342 | -1.500 | 0.599 | (0.307, 1.170) |
| >$99,999 | -0.385 | 0.372 | -1.035 | 0.680 | (0.328, 1.411) |
| **Health Insurance (Yes v. No)** | 1.138 | 0.845 | 1.348 | 3.121 | (0.596, 16.344) |
| **Cancer Treatments (None Reference)** |  |  |  |  |  |
| One | -0.295 | 0.417 | -0.708 | 0.744 | (0.329, 1.686) |
| Multiple | -0.401 | 0.437 | -0.918 | 0.670 | (0.284, 1.577) |
| **Urbanicity (Urban v. Rural)** | 0.298 | 0.339 | 0.880 | 1.348 | (0.694, 2.619) |
| **Marital Status (Married v. Unmarried)** | 0.190 | 0.239 | 0.794 | 1.209 | (0.757, 1.932) |
| **Age at Diagnosis (Years)** | 0.007 | 0.009 | 0.763 | 1.007 | (0.989, 1.025) |
| **Survey Year (2012 Reference)** |  |  |  |  |  |
| 2014 | -0.375 | 0.250 | -1.499 | 0.687 | (0.420, 1.123) |
| 2017 | 0.005 | 0.271 | 0.020 | 1.005 | (0.591, 1.711) |
| **Proportion of Life in US (%)** | 0.088 | 1.030 | 0.085 | 1.092 | (0.145, 8.225) |
| **Denied Insurance Coverage (Yes v. No)** | 0.298 | 0.345 | 0.863 | 1.347 | (0.685, 2.647) |
| **Cancer Hurt Finances (Yes v. No)** | 0.285 | 0.122 | 2.340 | 1.329 | (1.047, 1.687) |
| ***Note.*** Coefficients from logistic regression were used as starting values for ordinal regression. -1 and 1 were chosen as intercepts. | | | | | |
